# *Vibrio parahaemolyticus* Strains of Pandemic Serotypes Identified from Clinical and Environmental Samples from Jiangsu, China

**DOI:** 10.3389/fmicb.2016.00787

**Published:** 2016-05-31

**Authors:** Jingjiao Li, Feng Xue, Zhenquan Yang, Xiaoping Zhang, Dexin Zeng, Guoxiang Chao, Yuan Jiang, Baoguang Li

**Affiliations:** ^1^Animal Quarantine Laboratory, Jiangsu Entry-Exit Inspection and Quarantine BureauNanjing, China; ^2^Key Laboratory of Veterinary Biotechnology, Department of Animal Science, School of Agriculture and Biology, Shanghai JiaoTong UniversityShanghai, China; ^3^Jiangsu Key Laboratory of Zoonosis, School of Food Science and Engineering, Yangzhou UniversityYanghzou, China; ^4^Beijing Kemufeng Biopharmaceutical CompanyBeijing, China; ^5^Yangzhou Key Centre for Disease Control and PreventionYanghzou, China; ^6^Division of Molecular Biology, Center for Food Safety and Applied Nutrition, US Food and Drug AdministrationLaurel, MD, USA

**Keywords:** *Vibrio parahaemolyticus*, serotyping, MLST, virulence genes, TDH-related hemolysin (TRH), pandemic serotypes, epidemiology, phylogeny

## Abstract

*Vibrio parahaemolyticus* has emerged as a major foodborne pathogen in China, Japan, Thailand, and other Asian countries. In this study, 72 strains of *V. parahaemolyticus* were isolated from clinical and environmental samples between 2006 and 2014 in Jiangsu, China. The serotypes and six virulence genes including thermostable direct hemolysin (TDR) and TDR-related hemolysin (TRH) genes were assessed among the isolates. Twenty five serotypes were identified and O3:K6 was one of the dominant serotypes. The genetic diversity was assessed by multilocus sequence typing (MLST) analysis, and 48 sequence types (STs) were found, suggesting this *V. parahaemolyticus* group is widely dispersed and undergoing rapid evolution. A total of 25 strains of pandemic serotypes such as O3:K6, O5:K17, and O1:KUT were identified. It is worth noting that the pandemic serotypes were not exclusively identified from clinical samples, rather, nine strains were also isolated from environmental samples; and some of these strains harbored several virulence genes, which may render those strains pathogenicity potential. Therefore, the emergence of these “environmental” pandemic *V. parahaemolyticus* strains may poses a new threat to the public health in China. Furthermore, six novel serotypes and 34 novel STs were identified among the 72 isolates, indicating that *V. parahaemolyticus* were widely distributed and fast evolving in the environment in Jiangsu, China. The findings of this study provide new insight into the phylogenic relationship between *V. parahaemolyticus* strains of pandemic serotypes from clinical and environmental sources and enhance the MLST database; and our proposed possible O- and K- antigen evolving paths of *V. parahaemolyticus* may help understand how the serotypes of this dispersed bacterial population evolve.

## Introduction

*Vibrio parahaemolyticus* is a Gram-negative, halophilic bacterium that inhabits global coastal waters and rivers, and in seafood, such as fish and shellfish (Kelly and Stroh, 1988). *V. parahaemolyticus* was initially discovered in 1950 (Parveen et al., 2008; Letchumanan et al., 2015b). A novel serotype of O3:K6 clone was discovered in India in 1996 (Okuda et al., 1997), and since then, this clone and its serovariants have disseminated worldwide and become a pandemic clonal group (Ansaruzzaman et al., 2005; Quilici et al., 2005; Nair et al., 2007; Chao et al., 2009; Velazquez-Roman et al., 2013; Li W. et al., 2014; Flores-Primo et al., 2015).

*V. parahaemolyticus* can cause three major clinical syndromes: gastroenteritis, wound infections, and septicemia (Daniels et al., 2000), and is also considered as the causative agent of the most prevalent food poisoning in Asia since the outbreak in 1959 (Miyamoto et al., 1962). *V. parahaemolyticus* infections usually resulted from consumption of raw or undercooked seafood, mostly causing gastroenteritis (Miyamoto et al., 1969). Recently, *V. parahaemolyticus* has been identified as a major foodborne pathogen in food poisoning incidents in China, raising public health concern (Ma et al., 2014; Zhang et al., 2015).

*V. parahaemolyticus* was initially discovered in 1950 (Parveen et al., 2008). Traditionally, the identification of *V. parahaemolyticus* is performed by serological and biochemical tests. *V. parahaemolyticus* is classified by serotyping and the serotypes of *V. parahaemolyticus* are determined by the combination of somatic (O) antigens and capsular (K) antigens. There are 13 O serotypes and 71 K serotypes that have been reported (Iida et al., 1997; Nair et al., 2007; Chen et al., 2012). *V. parahaemolyticus* infections are associated with pathogenic strains of numerous serotypes (clinical); whereas non-pathogenic strains comprise an even greater variety of serotypes.

Serotypes are useful distinguishing features to identify clinical isolates (Jones et al., 2012), and serotyping has been widely used in epidemiological research and etiological diagnostics for many decades. However, the increasing genetic diversity such as emerging of new serotypes and STs among *V. parahaemolyticus* strains poses a challenge to this traditional way of strain identification and differentiation due to the high cost of the antisera and the potential ambiguity encountered during serotyping (Bogdanovich et al., 2003). In the last two decades, numerous DNA-based subtyping methods such as pulsed-field gel electrophoresis (PFGE; Wong et al., 1996), multilocus sequence typing (MLST; Gonzalez-Escalona et al., 2008), repetitive element PCR (Rep PCR; Wong and Lin, 2001), multilocus variable-number tandem-repeat analysis (MLVA; Kimura et al., 2008), clustered regularly interspaced short palindromic repeats (CRISPR; Sun et al., 2015), and microarray analysis (Li et al., 2015) have been developed to investigate the genetic diversity of outbreaks caused by *V. parahaemolyticus* and other foodborne pathogens (Li B. et al., 2014). Each of these subtyping methods has its advantages and disadvantages with respect to sensitivity, specificity, cost, and speed (Li et al., 2015). In general, MLST is the most commonly used method (Maiden, 2006; Nair et al., 2007; Gonzalez-Escalona et al., 2008), due to its high specificity, repeatability, and portability (Nair et al., 2007).

In this study, in an effort to assess the homogeneity and heterogeneity between the clinical and environmental *V. parahaemolyticus* isolates from Jiangsu Provence, an east coast area in China, where seafood is widely consumed, we used the traditional classification method, serotyping, to identify 72 *V. parahaemolyticus* strains from the food poisoning case samples and the environmental samples. The genetic diversity among *V. parahaemolyticus* strains were further assessed by MLST analysis and the presence of the virulence factors such as *tdh, trh, orf8, GS-PCR, PGS-PCR*, and *HU*-α*.* Furthermore, we analyzed the *V. parahaemolyticus* isolates by using eBURST and START (http://eburst.mlst.net) to investigate the relationship between clinical and environmental *V. parahaemolyticus* strains based on MLST databases. Additional information on genetic variation and the distribution of virulence genes among *V. parahaemolyticus* strains from various cities in Jiangsu Province would enrich the MLST database and epidemiological archive and be beneficial for the development of an efficient risk assessment of this common foodborne pathogen.

## Materials and methods

### *V. parahaemolyticus* strain identification

Seventy-two presumed *V. parahaemolyticus* isolates were collected from nine different cities in Jiangsu province of China between 2006 and 2014, including 21 clinical isolates from patients with food poisoning and 51 isolates from food samples. All strains were characterized according to GB 4789.7–2013 Chinese Food Safety Standards (http://www.foodmate.net). The *V. parahaemolyticus* strains were inoculated onto Vibrio culture plates (CHROMagar, Paris, France) and Thiosulphate Citrate Bile salt Sucrose [(TCBS) Beijing Land Bridge, China] culture plates and incubated at 37°C for 16–24 h. The colonies with typical contour were selected and characterized by VITEK automatic biochemical analyzer (Biomerieux, France).

### Serotyping

The serotype of *V. parahaemolyticus* strains was determined using two diagnostic kits; 11 antisera targeting the O1–O11 antigens and 71 antisera for the K antigens (Denka Seiken, Tokyo, Japan) and 11 antisera for O1–O13 antigens (Tianjin Biochip Corporation, Tianjin, China). Serotyping was carried out in accordance with the GB4789.7–2013 Chinese Food Safety Standard (http://www.foodmate.net). Single colonies were selected and plated onto the surface of 3% sodium chloride peptone agar plates, incubated at 37°C for 18 h. Bacterial suspension was obtained by washing the surface of agar with solution containing 3% NaCl and 5% glycerol.

O-antigen identification: The bacterial suspension was autoclaved at 121°C for 1.5 h followed by centrifugation at 12,000 g for 15 min. The pellets were washed two or three times with normal saline solution and centrifuged at 12,000 g for 15 min. The final suspension was used for O antiserum agglutination and normal saline solution was used as a negative control. If the result was negative, all the above steps were repeated; thereafter, the negative was considered as unknown antigen O.

K-antigen identification: Multi-serum against the K-antigen was added to the bacterial suspension. Positive colonies were selected for further analysis using individual K-antigen antiserum. Solution of NaCl (3%) was used as negative control.

### Identification of the *tdh, trh, orf8, GS-PCR, PGS-PCR*, and *HU*-α genes

Single colonies of *V. parahaemolyticus* strains were picked and inoculated into liquid culture medium containing 3% sodium chloride peptone. Cultures were incubated at 37°C for 16 h, followed by centrifugation at 12,000 g for 10 min. The genomic DNA was isolated using E.Z.N.A.TM kit (OMEGA, Beijing, China), and the concentrations of DNA were determined by spectrophotometry to ensure the OD260/OD280 was between 1.8 and 2.0.

The primers for the *tdh, trh, orf8, GS-PCR, PGS-PCR*, and *HU*-α genes were synthesized (Chao et al., 2009; Li W. et al., 2014; Table 1) by Sango Biotech Co., Ltd. (Shanghai, China). PCR reactions were performed in a volume of 25 μl containing 1 μl DNA (50 ng/μl), 12.5 μl 10 × PCR mix (Takara, Dalian, China), 1 μl of the forward and reverse primers of the virulence genes, and 9.5 μl of sterile distilled water. Strains ATCC33847 (*tdh*^+^*trh*^−^, isolated in US in 1973) and ATCC17802 (*tdh*^−^*trh*^+^, isolated in Japan in 1965) were used as positive controls. PCR reactions were performed under the following conditions: initial denaturation at 95°C for 2 min, followed by 30 cycles of denaturation at 95°C for 30 s, annealing at 50°C for 30 s, and elongation at 72°C for 1 min, and ending with elongation at 72°C for 5 min. PCR products (1 μl) were analyzed using Agilient 2100 analyzer (Waldbronn, Germany) to determine the PCR amplicon size.

**Table 1:** **Primers used for the detection of *V. parahaemolyticus* virulence genes and pandemic marker genes by PCR**.

**Gene**	**Sequence (5′—3′)**	**Amplicon (*bp*)**	**References**
*tdh-F^a^*	ATATCCATGTTGGCTGCATTC	531	Chao et al., 2009
*tdh-R*^b^	TTATTGTTGATGTTTACATTCAAAA		
*trh-F*	ATGAAACTAAAACTCTACTTTGC	553	Chao et al., 2009
*trh-R*	TTAATTTTGTGACATACATTCAT		
*orf8-F*	GTTCGCATACAGTTGAGG	700	Nasu et al., 2000
*orf8-R*	AAGTACACAGGAGTGAG		
*GS-PCR-F*	TAATGAGGTAGAAACA	651	Matsumoto et al., 2000
*GS-PCR-R*	ACGTAACGGGCCTACA		
*PGS-PCR-F*	TTCGTTTCGCGCCACAACT	235	Okura et al., 2004
*PGS-PCR-R*	TGCGGTGATTATTCGCGTCT		
*HU-α-F*	CGATAACCTATGAGAAGGGAAACC	474	Williams et al., 2004
*HU-α-R*	CTAGAAGGAAGAATTGATTGTCAAATAATG		

a *Forward primer*.

b *Reverse primer*.

### MLST

Seven housekeeping genes, *dnaE, gyrB, recA, dtdS, pntA, pyrC*, and *tnaA*, were selected as target genes in this study. The primers for these genes were adopted from MLST website (http://www.pubmlst.net). The MLST primers for *recA* gene failed in most strains in the current study, and new primers for *recA* gene were adopted from a previous study (Gonzalez-Escalona et al., 2008). All primers as shown in Table 2 were synthesized by Sango Biotech Co., Ltd. (China). Primers were diluted to 10 μM and stored at −20°C.

**Table 2:** **Primers of housekeeping genes used in MLST and PCR conditions in this study**.

**Locus**	**Primer sequence (5′— 3′)**	**Annealing (°C)**	**Extension (S)**	**Length (bp)**
*dnaE*	(F)tgtaaaacgacggccagtCGRATMACCGCTTTCGCCG	58	60	596
	(R)caggaaacagctatgaccGAKATGTGTGAGCTGTTTGC			
*gyrB*	(F)tgtaaaacgacggccagtGAAGGBGGTATTCAAGC	58	60	629
	(R)caggaaacagctatgaccGAGTCACCCTCCACWATGTA			
*dtdS*	(F)tgtaaaacgacggccagtTGGCCATAACGACATTCTGA	58	60	497
	(R)caggaaacagctatgaccGAGCACCAACGTGTTTAGC			
*pntA*	(F)tgtaaaacgacggccagtACGGCTACGCAAAAGAAATG	58	60	470
	(R)caggaaacagctatgaccTTGAGGCTGAGCCGATACTT			
*pyrC*	(F)tgtaaaacgacggccagtAGCAACCGGTAAAATTGTCG	58	60	533
	(R)caggaaacagctatgaccCAGTGTAAGAACCGGCACAA			
*tnaA*	(F)tgtaaaacgacggccagtTGTACGAAATTGCCACCAAA	58	60	463
	(R)caggaaacagctatgaccAATATTTTCGCCGCATCAAC			
*recA*	(F) GCTTCTGGTTGAGCTGGAGA	55	60	998
	(R) GACGAGAACAAACAGAAAGCG			

The underlined lower-cased letters represent common sequences that were adapted from M13 for more efficient sequencing.

### PCR amplification of housekeeping genes

To sequence the housekeeping genes, the PCR reaction volume was set at 50 μl. The components of PCR reactions as well as the parameters for each cycle were optimized for best performance. PCR products were visualized using Agilent electrophoresis and imaging system. The bidirectional DNA sequencing was conducted by Sango Biotech Co., Ltd.

### Allele and sequence analysis

The alignment of DNA sequences was performed by uploading each of the sequences of the seven housekeeping genes of the 72 *V. parahaemolyticus* strains to the MLST website (http://pubmlst.org/vparahaemolyticus/). If a novel allele or sequence was identified, the forward and reverse sequences were uploaded and submitted to the database administrator to obtain a serial number for the allele or sequence.

### Sequence analysis by eBURST v3.0 and START v2.0

The ST types of all the 72 strains were analyzed by eBURST v3.0 (http://eburst.mlst.net) to distinguish clonal complex (CC), group, and singleton STs. The most stringent definition was adopted to identify the homeotic complexes, each of which was defined by the presence of at least six or seven identical alleles. The single locus variant (SLV) was defined by the presence of a single allele difference between any two ST types, based on eBURST v3.0 analysis. The evolution of each allele and ST type was analyzed by using START v2.0 (http://pubmlst.org/software/analysis/start/).

## Results

*V. parahaemolyticus* colonies were round, translucent and purplish red on CHROMagar plates measuring 2–3 mm in diameter. They were round, translucent, and smooth green-colored colonies on TCBS plates. All 72 *V. parahaemolyticus* isolates were confirmed by VITEK biochemical analysis.

### Serotyping

There were 25 serotypes identified among the 72 *V. parahaemolyticus* isolates. The dominant serotypes were O3:K6 (*n* = 8) and O2:K28 (*n* = 8), followed by O1:KUT (*n* = 6), O5:K17 (*n* = 5), O5:KUT (*n* = 5), O1:K25 (*n* = 3), O4:K34 (*n* = 3), O10:KUT (*n* = 3), O2: KUT (*n* = 3), O1:K32 (*n* = 3). For the 21 clinical strains, nine serotypes were identifies where O3:K6 was the dominant serotype (*n* = 8), followed by O5:K17 (*n* = 5), O2:KUT (*n* = 3), and one strain of O1:K25, O11:K40, and O13:KUT (novel serotype). In addition, there was one strain (WX14115) that failed to react to either O or K antiserum (Table 3).

**Table 3:** **Serotypes, sequence types, allele profiles, and presence of virulence genes of the 72 *V. parahaemolyticus* strains**.

**Strain**	***dnaE***	***gyrB***	***recA***	***dtdS***	***pntA***	***pyrC***	***tnaA***	**ST**	**O**	**K**	***tdh***	***trh***	***GS-PCR***	***PGS-PCR***	***orf8***	***HU-α***
YZ0601	115	376	31	35	47	69	26	**968**^a^	2	28	−	−	+	+	+	+
YZ0602	81	212	75	84	63	26	47	**969**	8	39	−	−	+	+	−	−
YZ0603	28	4	82	88	63	187	1	**799**	5	UT	−	−	+	−	−	−
YZ0608	51	381	31	39	18	3	20	**988**	5	UT	−	−	+	+	+	+
YZ0612	28	4	245	88	63	187	1	**1108**	1	33	−	−	+	+	−	−
YZ0615	44	149	271	198	26	41	26	**989**	3	UT	−	−	+	+	−	+
YZ0618	104	252	272	29	26	306	23	**990**	10	UT	−	−	+	−	−	−
YZ0619	35	50	63	27	49	46	26	154	1	20	−	−	+	−	−	−
YZ0621	92	106	25	272	28	3	17	890	5	UT	−	−	+	−	−	−
YZ0625	51	29	77	13	60	8	33	356	1	33	−	−	+	−	−	−
YZ0626	131	147	60	136	90	27	23	276	1	UT	−	−	+	−	−	−
YZ0628	55	15	31	55	18	26	46	**991**	5	30	−	−	+	+	−	-
YZ0633	55	15	31	55	18	26	46	**991**	5	30	−	−	+	−	−	−
YZ0637	75	120	71	13	56	37	29	187	11	31	−	−	+	+	−	−
YZ0642	9	213	165	82	2	46	1	**992**	11	UT	−	−	+	+	+	+
YZ0646	275	376	31	35	47	69	26	**993**	2	28	−	−	−	−	−	−
YZ0647	60	104	210	126	28	226	159	**994**	4	34	−	−	+	+	−	−
YZ0650	30	276	75	171	61	73	57	**995**	2	28	−	−	+	+	−	−
YZ0652	270	371	273	120	23	238	26	**996**	5	UT	−	−	−	−	−	−
YZ0654	5	147	31	229	46	10	57	550	10	UT	−	−	+	+	−	−
YZ0656	35	50	63	27	49	46	26	154	1	20	−	−	+	−	−	−
YZ0658	175	43	274	253	194	190	9	997	2	28	−	−	+	+	−	−
YZ0659	167	2	109	293	28	307	105	**998**	2	25	−	−	+	+	−	−
YZ0663	270	371	275	120	23	238	26	**999**	5	UT	−	−	−	−	−	−
YZ0667	82	168	25	206	151	27	48	**1000**	2	28	−	−	+	−	−	−
YZ0668	175	43	274	253	194	190	9	**997**	2	28	−	−	+	−	−	−
YZ0673	69	92	69	114	54	71	24	212	1	UT	−	−	+	−	−	−
YZ0675	60	127	89	73	55	216	171	**1001**	1	32	−	−	+	−	−	−
YZ0676	69	92	69	267	54	71	24	**1002**	1	UT	−	−	−	−	−	−
YZ0684	60	217	31	18	106	150	26	**1003**	2	28	−	−	+	−	−	−
YZ0685	60	217	31	18	106	150	26	**1003**	2	28	−	−	+	+	−	−
YZ0686	60	104	210	126	28	226	159	**994**	4	34	−	−	+	−	−	−
YZ0688	60	217	31	18	106	150	26	**1003**	2	3	−	−	−	−	−	−
YZ0689	49	153	31	299	50	308	23	**1004**	10	UT	−	−	+	−	−	−
YZ0693	84	383	62	117	195	46	132	**1005**	11	UT	−	−	+	−	+	+
YZ0695	132	384	209	27	49	226	26	**1006**	1	25	−	−	−	−	−	−
YZ0696	69	92	69	114	54	71	24	212	1	32	−	−	+	−	−	−
YZ0697	44	89	31	73	46	309	86	**1007**	1	UT	−	−	+	−	−	−
HA08104	35	50	63	27	49	46	26	154	1	20	−	−	+	−	−	−
SH08108	131	147	60	136	90	27	23	276	1	25	−	−	+	−	−	−
WX08111	26	58	53	19	28	9	26	108	4	34	−	−	+	−	−	−
TC12100	31	115	22	12	4	91	68	1035	8	UT	−	−	+	−	−	−
CZ08101	270	371	273	120	23	238	26	996	5	UT	−	−	+	−	−	−
CS08103	49	153	31	299	50	308	23	**1004**	10	UT	−	−	+	−	−	−
YZ06115	84	383	62	117	195	46	132	**1005**	11	UT	−	−	+	−	−	−
KS08105	36	285	292	354	26	227	26	**1104**	4	9	−	−	+	−	−	−
RD08107	44	89	31	73	46	309	86	**1007**	1	32	−	−	+	−	−	−
SH08109	131	147	60	136	90	27	23	276	1	UT	−	−	−	−	−	−
TZ08110	283	82	31	355	53	45	13	**1105**	1	UT	−	−	+	+	−	−
YZ06114	35	352	151	47	26	325	1	**1106**	3	29	−	−	+	−	−	−
YZ06116	116	251	72	76	45	184	26	**1107**	4	42	−	−	−	+	−	−
WX1461	3	4	19	4	29	4	22	3	1	25	+	−	+	+	+	+
WX1465	3	4	19	4	29	4	22	3	3	6	+	−	+	+	+	+
WX1472	3	4	19	4	29	4	22	3	3	6	+	−	+	+	+	+
WX1475	80	252	160	179	26	10	23	**1109**	3	6	−	−	+	−	−	−
WX1477	3	4	19	4	29	4	22	3	3	6	+	−	+	+	+	+
WX1478	3	4	19	4	29	4	22	3	3	6	+	−	+	+	+	+
WX1483	14	30	141	78	4	37	13	332	4	UT	+	−	−	+	+	−
WX1486	3	4	19	4	29	4	22	3	3	6	+	−	+	+	−	+
WX1494	3	4	19	4	29	4	22	3	3	6	+	−	+	+	+	+
WX1498	31	366	264	339	26	45	24	**1110**	11	40	−	−	+	+	−	−
WX14102	173	406	73	47	4	116	227	**1111**	5	17	−	−	−	−	−	−
WX14103	284	343	293	191	23	326	132	**1112**	5	17	−	−	+	+	−	−
WX14105	35	43	38	21	31	35	37	79	5	17	−	+	+	−	−	−
WX14106	35	43	38	21	31	35	37	79	5	17	−	+	+	−	−	−
WX14107	34	4	216	151	201	327	33	**1113**	2	UT	−	−	−	+	−	−
WX14109	35	43	38	21	31	35	37	79	5	17	−	+	+	−	−	−
WX14113	110	407	70	76	78	328	148	**1114**	13	UT	−	+	−	−	−	−
WX14115	285	13	60	171	21	329	23	**1115**	UT	UT	−	−	+	+	+	+
WX14116	3	4	19	4	29	4	22	3	3	6	+	−	−	−	−	−
WX14118	31	106	135	74	37	212	54	564	2	UT	−	−	+	−	−	−
WX14119	31	106	135	74	37	212	54	564	2	UT	−	−	+	−	−	−

a *A bold-faced number refers a novel ST*.

Of the 25 serotypes, six were novel serotypes. Out of the six novel serotypes, five new serotypes were recovered from environmental samples and one was from clinical sample. Specifically, the five environmental strains are O2:K25, O4:K9, O4:K42, O8:K39, and O11:31, and the single clinical isolate is O13:KUT (Table 4). In the present study, 25 strains of pandemic serotypes were identified from Jiangsu Province, including 16 clinical strains and 9 environmental strains. Specifically, the clinical strains were serotypes O3:K6 (*n* = 8), O5:K17 (*n* = 5), and environmental strains were serotypes O1:KUT (*n* = 6), O1:K25 (*n* = 3), O3:K29 (*n* = 1), and O3:KUT (*n* = 1) (Table 3).

**Table 4:** ***V. parahaemolyticus* strains of pandemic serotypes and novel serotypes from clinical and environmental samples**.

**Strain**	**Sero- or sequence-type**			**Virulence factor**						**Source**
	**O**	**K**	**ST**	***tdh***	***trh***	***GS-PCR***	***PGS-PCR***	***orf8***	***HU-α***	
**PANDEMIC SEROTYPE**										
YZ0626	1	UT^a^	276	−	−	+	−	−	−	Environmental
YZ0673	1	UT	212	−	−	+	−	−	−	Environmental
YZ0676	1	UT	**1002**^b^	−	−	−	−	−	−	Environmental
YZ0697	1	UT	**1007**	−	−	+	−	−	−	Environmental
SH08109	1	UT	276	−	−	−	−	−	−	Environmental
TZ08110	1	UT	**1105**	−	−	+	+	−	−	Environmental
YZ06114	3	29	**1106**	−	−	+	−	−	−	Environmental
YZ0603	5	UT	**799**	−	−	+	−	−	−	Environmental
YZ0608	5	UT	**988**	−	−	+	+	+	+	Environmental
WX1461	1	25	3	+	−	+	+	+	+	Clinical
WX1465	3	6	3	+	−	+	+	+	+	Clinical
WX1472	3	6	3	+	−	+	+	+	+	Clinical
WX1477	3	6	3	+	−	+	+	+	+	Clinical
WX1478	3	6	3	+	−	+	+	+	+	Clinical
WX1486	3	6	3	+	−	+	+	−	+	Clinical
WX1494	3	6	3	+	−	+	+	+	+	Clinical
WX1475	3	6	**1109**	−	−	+	−	−	−	Clinical
WX1483	4	UT	332	+	−	−	+	+	−	Clinical
WX14102	5	17	**1111**	−	−	−	−	−	−	Clinical
WX14103	5	17	**1112**	−	−	+	+	−	−	Clinical
WX14105	5	17	79	−	+	+	−	−	−	Clinical
WX14106	5	17	79	−	+	+	−	−	−	Clinical
WX14109	5	17	79	−	+	+	−	−	−	Clinical
WX14113	**13**	**UT**	**1114**	−	+	−	−	−	−	Clinical
WX14115	UT	UT	**1115**	−	−	+	+	+	+	Clinical
**NOVEL SEROTYPE**										
YZ0659	**2**	**25**	**998**	−	−	+	+	−	−	Environmental
KS08105	**4**	**9**	**1104**	−	−	+	−	−	−	Environmental
YZ06116	**4**	**42**	**1107**		−	−	+	−	−	Environmental
YZ0602	**8**	**39**	**969**	−	−	+	+	−	−	Environmental
YZ0637	**11**	**31**	187	−	−	+	+	−	−	Environmental
WX14113	**13**	**UT**	**1114**	−	+	−	−	−	−	Clinical
WX14115	UT	UT	**1115**	−	−	+	+	+	+	Clinical

a *UT refers to untypable*.

b *Bold faced letters and number refer to a novel serotype or ST*.

### Identification of virulence factor genes *tdh, trh, GS-PCR, PGS-PCR, orf8*, and *HU*-α

The virulence genes in the 72 *V. parahaemolyticus* isolates were assessed by PCR. There were nine *tdh*^+^ strains and four *trh*^+^ strains, accounting for 61.9% (13/21) of the clinical strains. No strain was *tdh*^+^*trh*^+^. The nine *tdh*^+^ strains included seven O3:K6, one O1:K25 strain and one O4:KUT strain. Four *trh*^+^ strains included three O5:K20 and one O13:KUT (Table 3). The prevalence of the other four virulence genes, *GS-PCR, PGS-PCR, orf8, and HU*-α, are 81.9, 38.9, 16.9, and 18.9%, respectively. *GS-PCR* gene showed the highest prevalence and *orf8* gene showed the lowest prevalence for the four virulence genes in our collection (Table 3).

### MLST analysis

**Figure 1:**
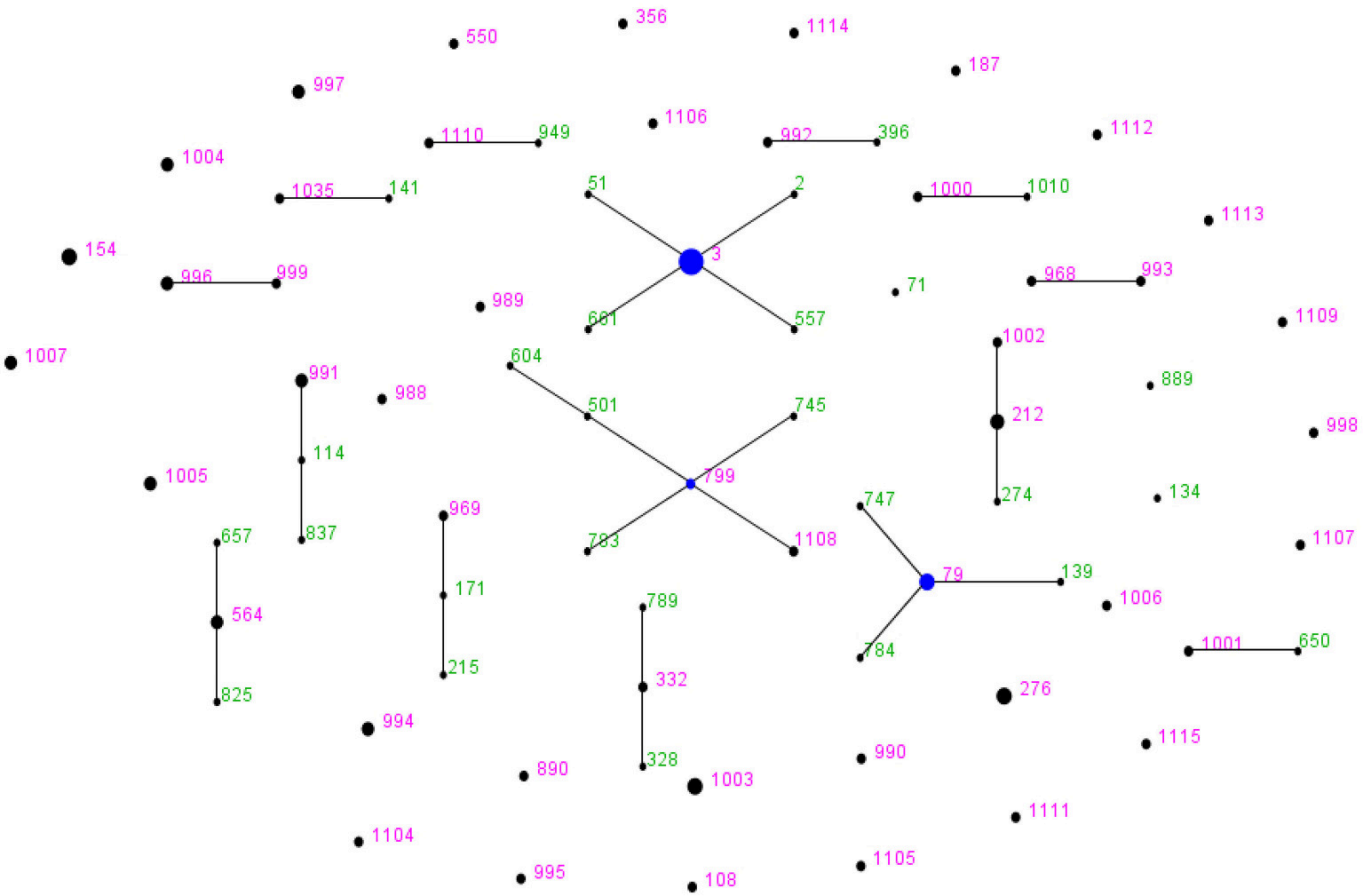
***V. parahaemolyticus* “population snapshot” of the 72 strains in this study created by using eBURST v3**. The 15 groups were defined using stringent criteria (5/7 shared alleles). The founder is labeled in blue, the number in green refers to the STs that have been existed in the query dataset; the number in pink represents the STs are among the reference and query dataset. The STs with SLVs to each other were shown connected by black lines. The sizes of the circles are relative to the numbers of strains in the ST.

All seven housekeeping genes were amplified in the *V. parahaemolyticus* strains using specific primers, and the PCR products were sequenced. **Seventy-two** *V. parahaemolyticus* strains were classified into 48 STs by MLST analysis. Of the 48 ST types, 34 were singleton and 14 were ST groups. Each singleton represented only one strain, while each ST group included two to eight strains (Figure 1). Twenty-one clinical strains were classified into 13 STs. The dominant ST was ST-3 with eight strains, followed by ST-79 and ST-564, each of which covered three strains. The remainder of STs was singletons. The MLST results indicated that *V. parahaemolyticus* strains showed genetic polymorphisms with much higher incidence rate in environmental strains than in clinical strains.

### Novel allele and STs

The ST composition of the 72 *V. parahaemolyticus* strains included 32 novel STs with 32 new allele values and 34 allele spectra, accounting for 79% of total STs. All of these newly identified allele values and allele spectra were submitted to the PubMLST database (http://pubmlst.org/vparahaemolyticus/) as shown in Table 3. There were 13 STs among the 21 clinical strains, including seven newly identified STs accounting 53.85% (7/13) of the ST types. There were 36 STs identified among the 51 environmental strains, including 27 newly identified STs, accounting 75% (27/36) of the STs. It appears that 13 new STs were formed through allele recombination, while the other 21 new STs were created by the newly identified housekeeping gene alleles which included several types of *dnaE* (*N* = 4), *gyrB* (*N* = 6), *recA* (*n* = 7), *dtdS* (*n* = 2), *pntA* (*n* = 3), *pyrC* (*n* = 9) genes, and one *tnaA* gene.

### Homologous allele complex and systematic development analysis

The system evolution diagram was plotted by eBURST v3 software, and 48 STs were divided into one clonal complex (CC), four double combinations (D), and 38 singletons (S). The CC identified in this study was the CC3 which covered seven strains of O3:K3 ST-3 and one strain of O3:KUT ST-3; these eight strains were epidemic strains from clinical samples. The D type included ST-799-ST-1108, ST-212-ST-1002, ST-996-ST-999, and ST-993-ST-968. The ST-1108, ST-1002, ST-996, ST-999, and ST-993 were newly identified in this study. Of the 38 singletons, 28 novel STs were identified in this study, which were genetically distant to the ST-3 and other STs (Table 3; Figure 1).

### Association of STs between serotypes and virulence genes

**Figure 2:**
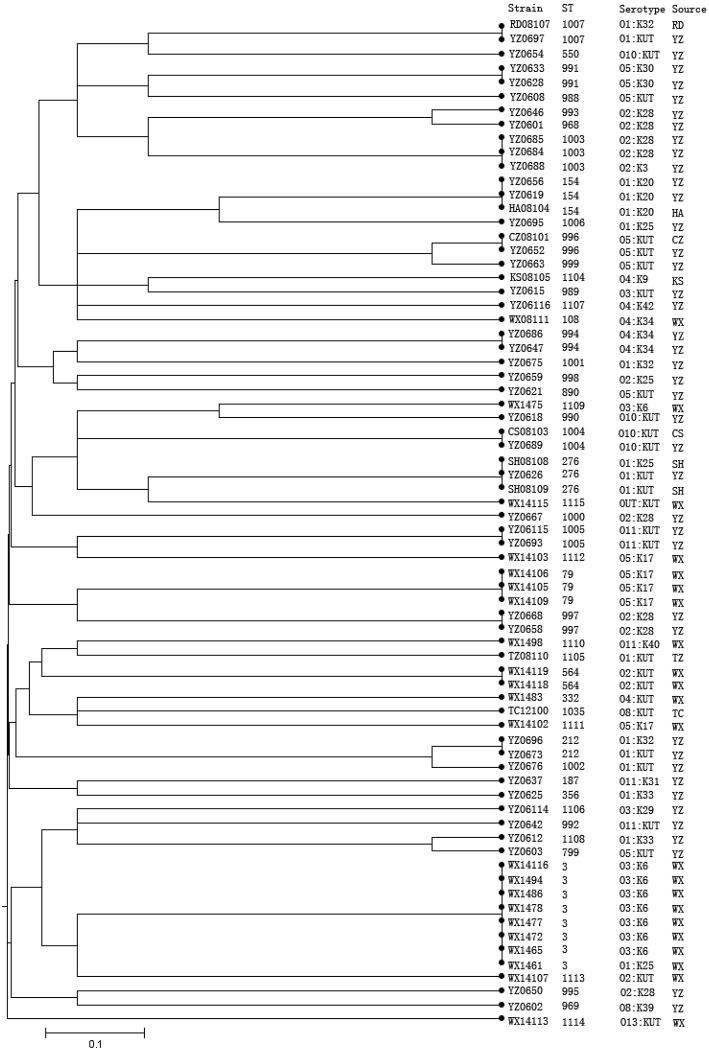
**Phylogenetic relationships of 72 *V. parahaemolyticus* strains based on the concatenated sequences of seven housekeeping genes**. The dendrogram is based on the UPGMA generated from allelic profiles of *V. parahaemolyticus* strains and was performed on START (http://pubmlst.org/software/analysis/start/) written by Jolley et al. (2001). Bioinformatics, 17, 1230–1231 (Jolley et al., 2001).

The strains with identical serotype usually showed similar STs or were clustered together, such as the majority serotype of O3:K6 in the present study belonging to ST-3; only one O3:KUT strain shared the ST-3 with O3:K6 strains. On the other hand, some strains with identical serotypes have different STs, such as strains of O1:KUT. These were identified as ST-1007, ST-212, and ST-276 (Figure 2).

There were nine *tdh*^+^ pathogenic strains identified in this study, including eight ST-3 strains (seven O3:K6 serotypes and one O1:K25 serotype), and one ST-332 strain (serotype O4:KUT). All of these 10 strains were epidemic strains from clinical samples. There were four *trh*^+^ strains, including three ST-79 (O5:K17) strains and one ST-1114 (O13:KUT) strain. All the four *trh*^+^ strains seemed to be epidemic strains (Table 4).

## Discussion

*V. parahaemolyticus* is the major foodborne pathogen. It is widely distributed with high survival and incidence rates, especially in the coastal areas of China (Su and Liu, 2007; Chao et al., 2009; Yan et al., 2015). Based on the epidemiological surveillance data from countries in Southeast Asia, *V. parahaemolyticus* infections have become a majority of foodborne pathogen (Pan et al., 1997; Wong et al., 1999; Obata et al., 2001; Liu et al., 2004; Cho et al., 2008; Letchumanan et al., 2014). In the present study, 25 serotypes and 48 STs were identified among the 72 *V. parahaemolyticus* isolates. Of the 25 serotypes and 48 STs, six additional novel serotypes and 34 novel STs were identified, indicating the *V. parahaemolyticus* population in Jiangsu Province was highly dispersed. The diversity in serotypes and STs of *V. parahaemolyticus*, especially environmental strains, is attributed to frequent recombination events in the pathogen but not by mutation (Gonzalez-Escalona et al., 2008). One example to support this is that the serotype of epidemic strains (O3:K6), which has been continuously evolving, resulted in 21 derivative serotypes of O3:K6 such as O4:K68, O1:K25, O1:KUT, O4:K12, and O5:K17 (Nair et al., 2007). All those strains demonstrated identical genotypes and molecular spectra, therefore the O3:K6 and its derivative are called O3K6 clones or pandemic strains. MLST analysis confirmed that O3:K6 and its derivatives belong to the same genetic lineage (Chowdhury et al., 2000, 2004; Matsumoto et al., 2000). Surprisingly, the 25 pandemic strains identified in this study were not exclusively from clinical samples (*n* = 16), rather, some strains were recovered from environmental samples (*n* = 9) as well.

Serotypes of O3:K6 and O5:K17 were the most common serotypes among the 21 clinical strains whereas some serotypes such as O11:K40, O4:K8, O2:KUT, and O13:KUT are rarely reported globally. It has been shown that serotype O1:KUT is closely related to pandemic strains and is seldom detected as environmental strains (Iida et al., 1997; Mahoney et al., 2010). However, in the present study, O1:KUT was identified as a common serotype with six isolates from environmental samples. More importantly, the six O1:KUT strains demonstrated extremely high genetic diversity with five different STs among the six strains (Table 4). Furthermore, of the five STs possessed by O1:KUT strains, three were novel STs, namely ST-1002, ST-1007, and ST-1105, suggesting these O1:KUT strains were highly dispersed and evolving rapidly in the environment. In addition, some of the O3:K6-specific pandemic markers such as *GS-PCR* and *PGS-PCR* were tested positive among most of these strains of pandemic serotypes. However, the pathogenicity of those strains needs to be further confirmed by recovering those strains from patients. Nevertheless, the risk posed to the public health in China by these “environmental” pandemic strains should not be overlooked.

The exact pathogenic mechanism of *V. parahaemolyticus* remains unclear, but the *tdh* and *trh* genes are considered the main pathogenic factors. Thermostable direct hemolysin (TDH), encoded by the *tdh* gene, manifests hemolytic, intestinal, and cardiac toxicities (Iida et al., 1997; Rosec et al., 2009; Raghunath, 2014). The *trh* gene is closely associated with the production of urease (Quilici et al., 2005). An epidemiological surveillance of *V. parahaemolyticus* in Northwest Mexico showed that up to 71.74% of the environmental isolates carried the *tdh* gene (De Jesus Hernandez-Diaz et al., 2015). However, numerous reports have shown that many pathogenic strains from patients were detected with neither of the two virulence factor genes, indicating more virulence factor genes are needed as markers for identification of pandemic *V. parahaemolyticus* strains (Garcia et al., 2009; Jones et al., 2012; Liu and Chen, 2015). Additionally, the type III secretion system (T3SS) of *V. parahaemolyticus* has been identified as a potential strain virulence factor (Park et al., 2004; Broberg et al., 2011).

*V. parahaemolyticus* O3:K6 serogroup has group-specific gene sequences in the *toxRs* operon and *orf8*, one of the 10 known open reading frames (ORFs) which is unique to the O3:K6 filamentous phage f237. The *toxRs* and *orf8* genes have been used as genetic markers to differentiate O3:K6 from other serogroups (Matsumoto et al., 2000; Nasu et al., 2000). Additionally, the *HU*-αORF, a specific biomarker for pandemic strain, which has a C-terminal amino acid sequence different from those of other strains of *V. parahaemolyticus*, was used to identify O3:K6 and other serotypes, such as O1:K25, O1:KUT, and O4:K68 (Matsumoto et al., 2000; Williams et al., 2004).

It has been reported that *V. parahaemolyticus* that lacked the *tdh* and *trh* genes were pathogenic in a study using mice (Rosec et al., 2009). In this study, most of the clinical strains (13/21) were negative for the *tdh* and *trh* genes, but possessed at least one of the four other virulence genes, *GS-PCR, PGS-PCR, orf8*, and *HU*-α. For instance, two *tdh*^−^*trh*^−^ strains, OUT:KUT (WX14115, clinical) and O5:KUT (YZ0608, environmental) were positive for the other four virulence genes (*GS-PCR, PGS-PCR, orf8*, and *HU*-α). Using a panel of six virulence genes as pathogenic markers, almost all the O3:K6 strains (except for two strains, WX1475 and WX14116) were positive for at least four of the six virulence genes, and only one clinical strain of O5:K17 (WX14102) was negative for all the six virulence genes among the 21 clinical strains. Thus, the results of this panel of virulence genes may more closely reflect the pathogenicity potential of those strains.

A high percentage (90.3%, 65/72) of strains were positive for at least one of the six virulence genes, *tdh, trh, GS-PCR, PGS-PCR, orf8*, and *HU*-α*.* Out of the six virulence genes, the *GS-PCR* gene showed the highest prevalence 81.9% (59/72). This result is very different from other investigators in China, where GS-PCR gene was seldom detected from environmental samples (Alam et al., 2009; Chao et al., 2009; Zhang et al., 2013). This difference might be a reflection of the genetic diversity between the indigenous isolates in this area (Jiangsu, China) and other areas or an artifact caused by sampling difference.

The *GS-PCR* gene has been shown to be a specific for genetic marker for the identification of pandemic *V. parahaemolyticus* strains (Li W. et al., 2014; Pazhani et al., 2014). The *trh* gene showed the lowest prevalence 5.6% (4/72), which is consistent with the results from other Asia countries (Alam et al., 2009; Chao et al., 2009; Zhang et al., 2013, 2015; Letchumanan et al., 2015a). Of note is that a number of different serotypes of *tdh*^−^*trh*^−^ strains (such as O28:K28, O5:KUT, and O11:KUT) were positive for at least three of the four virulence genes, *GS-PCR, PGS-PCR, orf8*, and *HU*-α (Table 3). Although these strains were isolated from the environment, it is quite possible for a non-pathogenic strain to gain pathogenicity potential after acquiring several virulence genes from pathogenic strains as indicated by the evolving path of the pandemic strain O3:K6 (Nair et al., 2007).

In this study, there were 34 new STs out of the 48 ST types, and the newly defined gene spectra accounted for 70.83%, suggesting this *V. parahaemolyticus* group is highly dispersed. The cluster diagram of MLST analysis showed that *V. parahaemolyticus* strains in Jiangsu area demonstrated a higher level of polymorphisms within environmental strains than clinical strains. ST-3 was the dominant ST among the 21 clinical strains and all ST-3 strains (*n* = 7) belonged to pandemic O3:K6 serotype, which is in agreement with previous reports from China and other countries (Gonzalez-Escalona et al., 2008).

It is worth noting in this study that there were a number of cases in which strains with identical serotype were classified into different STs; while strains with an identical ST possessed different serotypes. For instance, several strains of O1:KUT serotype were subtyped as ST1007, ST212, and ST276 by MLST; and the same ST strains, such as ST-3 strains, included O3:K6 and O3:KUT serotypes, whereas ST-276 strains included O1K:25 and O1K:KUT serotypes (Table 5). This observation not only indicates the advantages and limitations of serotyping and MLST analysis, but also implies that frequent mutation and/or recombination occur on the O- and K-antigens on the chromosome.

**Table 5:**
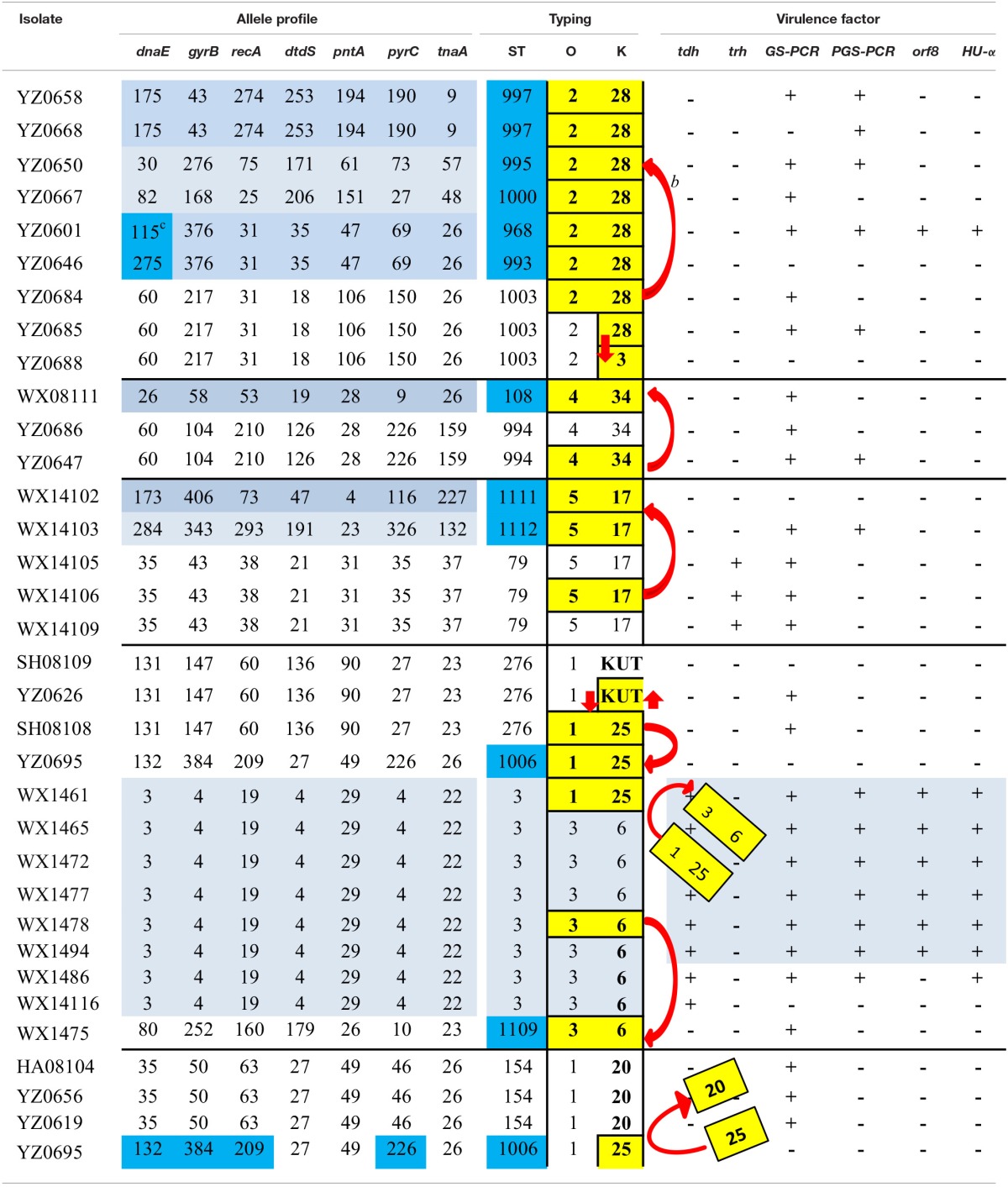
**Possible recombination scenerios of reprentive strains of *V. parahaemolyticus* that share a serotype or a ST^a^**.

*^a^ST, sequence type; O, O-antigen; K, K-antigen*.

*^b^An arrow refers to an O- and K-antigen or K antigen exchange between strains of different serotypes or STs*.

*^c^A blue-highlighted number indicates a new allelic gene*.

In this study, we used serotyping and MLST methods to differentiate the *V. parahaemolyticus* isolates and assessed the presence of six virulence factors, *tdh, trh, orf8, GS-PCR, PGS-PCR*, and *HU*-α*.* A total of 25 serotypes and 48 STs were identified among the 72 *V. parahaemolyticus* isolates; six novel serotypes and 34 novel STs were identified; and highly variable profiles of six virulence factor genes were detected among the isolates, suggesting this *V. parahaemolyticus* group was a highly dispersed group and was evolving rapidly. This information not only can enrich the MLST database but also can serve as a valuable set of matrices to trace the gene mutation and HGT (or recombination) among the *V. parahaemolyticus* population.

Data from Table 5 indicate that the O- and K-antigens move together more often than to move independently; and the K-antigen is more likely to be swapped than O-antigen. For example, the ST-3 strains included, in addition to the seven O3:K6 strains, a single strain of O1:K25 (WX1461). TheO1:K25 strain (belonged to ST-3) can serve as an example for O- and K-antigen exchange between serotypes O3:K6 and O1:K25, i.e., the O3:K6 antigens of strain WX1461 were replaced by O1:K25 antigens as evidenced by its identical allele and virulence gene profiles with seven O3:K6 (ST-3) strains. Another example is WX1475 which is serotype O3:K6 but was subtyped as ST-1109 by MLST based on different allele and virulent gene profiles than with those other O3:K6 serotype strains (Table 5, lower middle). The third example is that strains with different STs acquired the O2:K28 antigens from a strain with different ST (ST-1003; Top of Table 5). Specifically, a serotype O2:K28 isolate (YZ0864) was subtyped as ST-1003 with another serotype O2:K28 isolate (YZ0685).

In this study, there were eight strains with serotype O2:K28 but were differentiated into six different STs, based on the high discriminatory power of MLST (Table 5). Serotype O2:K28 was initially found in 1977 (Libinzon et al., 1977) and is generally considered as an environmental strain (Martinez-Urtaza et al., 2004). Drastic differences were found between the results derived from the two subtyping methods. It is hard to pinpoint the exact cause(s) that led to the differences in allele profiles among those isolates during their evolution path. However, a plausible scenario is that the O- and K-antigens of the six strains (O2:K28) of different STs might have been acquired from strains such as YZ0684 (O2:K28; ST-1003) over years (Table 5).

Similar virulence gene profiles exist between the O2:K28 strains of ST-1003 and the six different STs strains (lacking *tdh* and *trh* genes but are positive for *GS-PCR*) seem to corroborate this notion (Table 5). In addition to the genetic evidence generated in this study, our hypothesis is also supported by the findings on bacterial antigens, virulence genes, and genetic traits of *V. parahaemolyticus* (Chowdhury et al., 2000; Gonzalez-Escalona et al., 2008; Chao et al., 2009; Mala et al., 2016). As shown in Table 5, serotype conversion (from one serotype to a different serotype) occurred more frequently than ST change (from one ST to a different ST) among the listed strains, whose serotype was shared by strains of different STs or whose ST was shared by strains of different serotypes. It seemed that O- and K-antigen conversion occurred simultaneously more often than independently, suggesting that the O- and K-antigens are actively evolving and the two antigens are mostly moving together by horizontal gene transfer (HGT) (or recombination). This presumption is well in agreement with the recent findings on *V. parahaemolyticus* genomic evolution that HGT is 10–1000 times more attributable than single nucleotide variants to genome diversification. This may be the underlying drive that is responsible for the high diversity among the *V. parahaemolyticus* studied. This hypothesis is consistent with other researchers on the diversity and pathogenicity of *V. parahaemolyticus* (Chowdhury et al., 2000; Gonzalez-Escalona et al., 2008; Chao et al., 2009; Mala et al., 2016). Furthermore, other studies on K-antigen and comparative genomic analysis of *V. parahaemolyticus* (Chen et al., 2010, 2011), have demonstrated that the O- and K-antigens are at two adjacent loci on chromosome II and, thus the O- and K-antigens could be swapped via a single recombination event to create both novel O- and K-antigens (Chen et al., 2010). Moreover, the human upper intestine is believed to be a particularly suitable niche for the intra- and inter-specific lateral transfer of genetic material necessary to enhance bacterial pathogenicity (Larocque et al., 2005; Okada et al., 2009; Hasan et al., 2010; Wang et al., 2011). Therefore, our model is supported not only by multiple lines of genetic evidence from *V. parahaemolyticus*, but also by the presence of suitable ecological niche that can facilitate HGT. With this notion, we can better interpret the scenarios we encountered here, i.e., the pandemic *V. parahaemolyticus* strains recovered not only from patients, but also from nine “environmental” seafood samples; and the high genetic diversity among the 72 isolates.

In summary, *V. parahaemolyticus* in Jiangsu, China, were highly dispersed and widely distributed in the environment. In light of that *V. parahaemolyticus* has become one of the major foodborne pathogens in China in recent years (Li Y. et al., 2014; Qi et al., 2016), the new threat to the public health posed by these “environmental” pandemic strains should not be overlooked. T. The findings of this study provide new insight into the phylogenic relationship between *V. parahaemolyticus* strains of pandemic serotypes from clinical and environmental sources; the information on the genetic diversity among isolates enriches the MLST database; and our proposed possible O- and K- antigen evolving paths of *V. parahaemolyticus* may help understand how the serotypes of this dispersed bacterial population evolve. Our findings also underscores the necessity for more epidemiological studies and more comprehensive surveillances on *V. parahaemolyticus* in order to efficiently prevent the diseases caused by this organism.

## Author contributions

JL performed the experiments. YJ, FX, and BL conceived and designed the study. XZ, DZ, JL, FX, and BL analyzed the data. ZY and GC provided the isolates. JL, YJ, FX, and BL wrote the manuscript. All the authors reviewed the manuscript.

### Conflict of interest statement

The authors declare that the research was conducted in the absence of any commercial or financial relationships that could be construed as a potential conflict of interest.

## References

[B1] Alam M. Chowdhury W. B. Bhuiyan N. A. Islam A. Hasan N. A. Nair G. B.. (2009). Serogroup, virulence, and genetic traits of *Vibrio parahaemolyticus* in the estuarine ecosystem of Bangladesh. Appl. Environ. Microbiol. 75, 6268–6274. 10.1128/AEM.00266-09 19684167 PMC2753069

[B2] Ansaruzzaman M. Lucas M. Deen J. L. Bhuiyan N. A. Wang X. Y. Safa A.. (2005). Pandemic serovars (O3:K6 and O4:K68) of *Vibrio parahaemolyticus* associated with diarrhea in Mozambique: spread of the pandemic into the African continent. J. Clin. Microbiol. 43, 2559–2562. 10.1128/JCM.43.6.2559-2562.2005 15956363 PMC1151933

[B3] Bogdanovich T. Carniel E. Fukushima H. Skurnik M. (2003). Use of O-antigen gene cluster-specific PCRs for the identification and O-genotyping of *Yersinia pseudotuberculosis* and *Yersinia pestis*. J. Clin. Microbiol. 41, 5103–5112. 10.1128/JCM.41.11.5103-5112.2003 14605146 PMC262526

[B4] Broberg C. A. Calder T. J. Orth K. (2011). *Vibrio parahaemolyticus* cell biology and pathogenicity determinants. Microbes Infect. 13, 992–1001. 10.1016/j.micinf.2011.06.013 21782964 PMC3384537

[B5] Chao G. Jiao X. Zhou X. Yang Z. Huang J. Pan Z.. (2009). Serodiversity, pandemic O3:K6 clone, molecular typing, and antibiotic susceptibility of foodborne and clinical *Vibrio parahaemolyticus* isolates in Jiangsu, China. Foodborne Pathog. Dis. 6, 1021–1028. 10.1089/fpd.2009.0295 19630509

[B6] Chen M. Guo D. Wong H. C. Zhang X. Liu F. Chen H.. (2012). Development of O-serogroup specific PCR assay for detection and identification of *Vibrio parahaemolyticus*. Int. J. Food Microbiol. 159, 122–129. 10.1016/j.ijfoodmicro.2012.08.012 23072697

[B7] Chen Y. Dai J. Morris J. G. Jr. Johnson J. A. (2010). Genetic analysis of the capsule polysaccharide (K antigen) and exopolysaccharide genes in pandemic *Vibrio parahaemolyticus* O3:K6. BMC Microbiol. 10:274. 10.1186/1471-2180-10-274 21044320 PMC2987987

[B8] Chen Y. Stine O. C. Badger J. H. Gil A. I. Nair G. B. Nishibuchi M.. (2011). Comparative genomic analysis of *Vibrio parahaemolyticus*: serotype conversion and virulence. BMC Genomics 12:294. 10.1186/1471-2164-12-294 21645368 PMC3130711

[B9] Cho S. H. Shin H. H. Choi Y. H. Park M. S. Lee B. K. (2008). Enteric bacteria isolated from acute diarrheal patients in the Republic of Korea between the year 2004 and 2006. J. Microbiol. 46, 325–330. 10.1007/s12275-008-0015-4 18604503

[B10] Chowdhury N. R. Chakraborty S. Ramamurthy T. Nishibuchi M. Yamasaki S. Takeda Y.. (2000). Molecular evidence of clonal *Vibrio parahaemolyticus* pandemic strains. Emerg. Infect. Dis. 6, 631–636. 10.3201/eid0606.000612 11076722 PMC2640929

[B11] Chowdhury N. R. Stine O. C. Morris J. G. Nair G. B. (2004). Assessment of evolution of pandemic *Vibrio parahaemolyticus* by multilocus sequence typing. J. Clin. Microbiol. 42, 1280–1282. 10.1128/JCM.42.3.1280-1282.2004 15004094 PMC356825

[B12] Daniels N. A. MacKinnon L. Bishop R. Altekruse S. Ray B. Hammond R. M.. (2000). *Vibrio parahaemolyticus* infections in the United States, 1973–1998. J. Infect. Dis. 181, 1661–1666. 10.1086/315459 10823766

[B13] De Jesus Hernandez-Diaz L. Leon-Sicairos N. Velazquez-Roman J. Flores-Villasenor H. Guadron-Llanos A. M. Martinez-Garcia J. J.. (2015). A pandemic *Vibrio parahaemolyticus* O3:K6 clone causing most associated diarrhea cases in the Pacific Northwest coast of Mexico. Front. Microbiol. 6:221. 10.3389/fmicb.2015.00221 25852677 PMC4371747

[B14] Flores-Primo A. Pardio-Sedas V. T. Lopez-Hernandez K. Lizarraga-Partida L. Uscanga-Serrano R. (2015). [Growth and survival of total and pathogenic *Vibrio parahaemolyticus* in American oyster (*Crassostrea virginica*) under cold storage]. Salud Publica Mex. 57, 211–218. 26302123

[B15] Garcia K. Torres R. Uribe P. Hernandez C. Rioseco M. L. Romero J.. (2009). Dynamics of clinical and environmental *Vibrio parahaemolyticus* strains during seafood-related summer diarrhea outbreaks in southern Chile. Appl. Environ. Microbiol. 75, 7482–7487. 10.1128/AEM.01662-09 19801458 PMC2786416

[B16] Gonzalez-Escalona N. Martinez-Urtaza J. Romero J. Espejo R. T. Jaykus L. A. Depaola A. (2008). Determination of molecular phylogenetics of *Vibrio parahaemolyticus* strains by multilocus sequence typing. J. Bacteriol. 190, 2831–2840. 10.1128/JB.01808-07 18281404 PMC2293261

[B17] Hasan N. A. Grim C. J. Haley B. J. Chun J. Alam M. Taviani E.. (2010). Comparative genomics of clinical and environmental *Vibrio mimicus*. Proc. Natl. Acad. Sci. U.S.A. 107, 21134–21139. 10.1073/pnas.1013825107 21078967 PMC3000290

[B18] Iida T. Suthienkul O. Park K. S. Tang G. Q. Yamamoto R. K. Ishibashi M.. (1997). Evidence for genetic linkage between the ure and trh genes in *Vibrio parahaemolyticus*. J. Med. Microbiol. 46, 639–645. 10.1099/00222615-46-8-639 9511811

[B19] Jolley K. A. Feil E. J. Chan M. S. Maiden M. C. (2001). Sequence type analysis and recombinational tests (START). Bioinformatics 17, 1230–1231. 10.1093/bioinformatics/17.12.1230 11751234

[B20] Jones J. L. Ludeke C. H. Bowers J. C. Garrett N. Fischer M. Parsons M. B.. (2012). Biochemical, serological, and virulence characterization of clinical and oyster *Vibrio parahaemolyticus* isolates. J. Clin. Microbiol. 50, 2343–2352. 10.1128/JCM.00196-12 22535979 PMC3405591

[B21] Kelly M. T. Stroh E. M. (1988). Occurrence of *Vibrionaceae* in natural and cultivated oyster populations in the Pacific Northwest. Diagn. Microbiol. Infect. Dis. 9, 1–5. 10.1016/0732-8893(88)90054-5 3370928

[B22] Kimura B. Sekine Y. Takahashi H. Tanaka Y. Obata H. Kai A.. (2008). Multiple-locus variable-number of tandem-repeats analysis distinguishes *Vibrio parahaemolyticus* pandemic O3:K6 strains. J. Microbiol. Methods 72, 313–320. 10.1016/j.mimet.2007.12.014 18258320

[B23] Larocque R. C. Harris J. B. Dziejman M. Li X. Khan A. I. Faruque A. S.. (2005). Transcriptional profiling of *Vibrio cholerae* recovered directly from patient specimens during early and late stages of human infection. Infect. Immun. 73, 4488–4493. 10.1128/IAI.73.8.4488-4493.2005 16040959 PMC1201252

[B24] Letchumanan V. Chan K. G. Lee L. H. (2014). *Vibrio parahaemolyticu*s: a review on the pathogenesis, prevalence, and advance molecular identification techniques. Front. Microbiol. 5:705. 10.3389/fmicb.2014.00705 25566219 PMC4263241

[B25] Letchumanan V. Pusparajah P. Tan L. T. Yin W. F. Lee L. H. Chan K. G. (2015a). Occurrence and antibiotic resistance of *Vibrio parahaemolyticus* from Shellfish in Selangor, Malaysia. Front. Microbiol. 6:1417. 10.3389/fmicb.2015.01417 26697003 PMC4678184

[B26] Letchumanan V. Yin W. F. Lee L. H. Chan K. G. (2015b). Prevalence and antimicrobial susceptibility of *Vibrio parahaemolyticus* isolated from retail shrimps in Malaysia. Front. Microbiol. 6:33. 10.3389/fmicb.2015.00033 25688239 PMC4311705

[B27] Li B. Jackson S. A. Gangiredla J. Wang W. Liu H. Tall B. D.. (2015). Genomic evidence reveals numerous *Salmonella enterica* serovar Newport reintroduction events in Suwannee watershed irrigation ponds. Appl. Environ. Microbiol. 81, 8243–8253. 10.1128/AEM.02179-15 26386063 PMC4644655

[B28] Li B. Vellidis G. Liu H. Jay-Russell M. Zhao S. Hu Z.. (2014). Diversity and antimicrobial resistance of *Salmonella enterica* isolates from surface water in Southeastern United States. Appl. Environ. Microbiol. 80, 6355–6365. 10.1128/AEM.02063-14 25107969 PMC4178646

[B29] Li W. Mei L. Tang Z. Yang X. Li X. Pei X.. (2014). [Analysis of molecular features of clinical *Vibrio parahaemolyticus* strains in China]. Zhonghua Yu Fang Yi Xue Za Zhi 48, 44–52. 24713290

[B30] Li Y. Xie X. Shi X. Lin Y. Qiu Y. Mou J.. (2014). *Vibrio parahaemolyticus*, Southern Coastal Region of China, 2007-2012. Emerg. Infect. Dis. 20, 685–688. 10.3201/eid2004.130744 24655369 PMC3966377

[B31] Libinzon A. E. Demina A. I. Kulov G. I. Shestialtynova I. S. Manuk'ian G. V. (1977). [*Halophilic vibrios* isolated from the Sea of Azov]. Zh. Mikrobiol. Epidemiol. Immunobiol. 6, 77–80. 899437

[B32] Liu M. Chen S. (2015). A novel adhesive factor contributing to the virulence of *Vibrio parahaemolyticus*. Sci. Rep. 5:14449. 10.1038/srep14449 26399174 PMC4585867

[B33] Liu X. Chen Y. Wang X. Ji R. (2004). [Foodborne disease outbreaks in China from 1992 to 2001 national foodborne disease surveillance system]. Wei Sheng Yan Jiu 33, 725–727. 15727189

[B34] Ma C. Deng X. Ke C. He D. Liang Z. Li W.. (2014). Epidemiology and etiology characteristics of foodborne outbreaks caused by *Vibrio parahaemolyticus* during 2008-2010 in Guangdong province, China. Foodborne Pathog. Dis. 11, 21–29. 10.1089/fpd.2013.1522 24138080

[B35] Mahoney J. C. Gerding M. J. Jones S. H. Whistler C. A. (2010). Comparison of the pathogenic potentials of environmental and clinical *Vibrio parahaemolyticus* strains indicates a role for temperature regulation in virulence. Appl. Environ. Microbiol. 76, 7459–7465. 10.1128/AEM.01450-10 20889774 PMC2976215

[B36] Maiden M. C. (2006). Multilocus sequence typing of bacteria. Annu. Rev. Microbiol. 60, 561–588. 10.1146/annurev.micro.59.030804.121325 16774461

[B37] Mala W. Alam M. Angkititrakul S. Wongwajana S. Lulitanond V. Huttayananont S.. (2016). Serogroup, virulence, and molecular traits of *Vibrio parahaemolyticus* isolated from clinical and cockle sources in northeastern Thailand. Infect. Genet. Evol. 39, 212–218. 10.1016/j.meegid.2016.01.006 26773828

[B38] Martinez-Urtaza J. Lozano-Leon A. Depaola A. Ishibashi M. Shimada K. Nishibuchi M.. (2004). Characterization of pathogenic *Vibrio parahaemolyticus* isolates from clinical sources in Spain and comparison with Asian and North American pandemic isolates. J. Clin. Microbiol. 42, 4672–4678. 10.1128/JCM.42.10.4672-4678.2004 15472326 PMC522348

[B39] Matsumoto C. Okuda J. Ishibashi M. Iwanaga M. Garg P. Rammamurthy T.. (2000). Pandemic spread of an O3:K6 clone of *Vibrio parahaemolyticus* and emergence of related strains evidenced by arbitrarily primed PCR and toxRS sequence analyses. J. Clin. Microbiol. 38, 578–585. 10655349 10.1128/jcm.38.2.578-585.2000 PMC86152

[B40] Miyamoto Y. Kato T. Obara Y. Akiyama S. Takizawa K. Yamai S. (1969). *In vitro* hemolytic characteristic of *Vibrio parahaemolyticus*: its close correlation with human pathogenicity. J. Bacteriol. 100, 1147–1149. 5391048 10.1128/jb.100.2.1147-1149.1969 PMC250216

[B41] Miyamoto Y. Nakamura K. Takizawa K. (1962). Seasonal distribution of *oceanomonas* spp., halophilic bacteria, in the coastal sea. Its significance in epidemiology and marine industry. Jpn. J. Microbiol. 6, 141–158.

[B42] Nair G. B. Ramamurthy T. Bhattacharya S. K. Dutta B. Takeda Y. Sack D. A. (2007). Global dissemination of *Vibrio parahaemolyticus* serotype O3:K6 and its serovariants. Clin. Microbiol. Rev. 20, 39–48. 10.1128/CMR.00025-06 17223622 PMC1797631

[B43] Nasu H. Iida T. Sugahara T. Yamaichi Y. Park K. S. Yokoyama K.. (2000). A filamentous phage associated with recent pandemic *Vibrio parahaemolyticus* O3:K6 strains. J. Clin. Microbiol. 38, 2156–2161. 10834969 10.1128/jcm.38.6.2156-2161.2000 PMC86752

[B44] Obata H. Kai A. Morozumi S. (2001). [The trends of *Vibrio parahaemolyticus* foodborne outbreaks in Tokyo: 1989-2000]. Kansenshogaku Zasshi 75, 485–489. 10.11150/kansenshogakuzasshi1970.75.485 11494566

[B45] Okada N. Iida T. Park K. S. Goto N. Yasunaga T. Hiyoshi H.. (2009). Identification and characterization of a novel type III secretion system in trh-positive *Vibrio parahaemolyticus* strain TH3996 reveal genetic lineage and diversity of pathogenic machinery beyond the species level. Infect. Immun. 77, 904–913. 10.1128/IAI.01184-08 19075025 PMC2632016

[B46] Okuda J. Ishibashi M. Hayakawa E. Nishino T. Takeda Y. Mukhopadhyay A. K.. (1997). Emergence of a unique O3:K6 clone of *Vibrio parahaemolyticus* in Calcutta, India, and isolation of strains from the same clonal group from Southeast Asian travelers arriving in Japan. J. Clin. Microbiol. 35, 3150–3155. 9399511 10.1128/jcm.35.12.3150-3155.1997 PMC230139

[B47] Okura M. Osawa R. Iguchi A. Takagi M. Arakawa E. Terajima J.. (2004). PCR-based identification of pandemic group *Vibrio parahaemolyticus* with a novel group-specific primer pair. Microbiol. Immunol. 48, 787–790. 10.1111/j.1348-0421.2004.tb03596.x 15502414

[B48] Pan T. M. Wang T. K. Lee C. L. Chien S. W. Horng C. B. (1997). Food-borne disease outbreaks due to bacteria in Taiwan, 1986 to 1995. J. Clin. Microbiol. 35, 1260–1262. 9114420 10.1128/jcm.35.5.1260-1262.1997 PMC232742

[B49] Park K. S. Ono T. Rokuda M. Jang M. H. Okada K. Iida T.. (2004). Functional characterization of two type III secretion systems of *Vibrio parahaemolyticus*. Infect. Immun. 72, 6659–6665. 10.1128/IAI.72.11.6659-6665.2004 15501799 PMC523034

[B50] Parveen S. Hettiarachchi K. A. Bowers J. C. Jones J. L. Tamplin M. L. McKay R.. (2008). Seasonal distribution of total and pathogenic *Vibrio parahaemolyticus* in Chesapeake Bay oysters and waters. Int. J. Food Microbiol. 128, 354–361. 10.1016/j.ijfoodmicro.2008.09.019 18963158

[B51] Pazhani G. P. Bhowmik S. K. Ghosh S. Guin S. Dutta S. Rajendran K.. (2014). Trends in the epidemiology of pandemic and non-pandemic strains of *Vibrio parahaemolyticus* isolated from diarrheal patients in Kolkata, India. PLoS Negl. Trop. Dis. 8:e2815. 10.1371/journal.pntd.0002815 24786538 PMC4006737

[B52] Qi X. L. Wang H. X. Bu S. R. Xu X. G. Wu X. Y. Lin D. F. (2016). Incidence rates and clinical Symptoms of *Salmonella, Vibrio parahaemolyticus*, and *Shigella* infections in China, 1998-2013. J. Infect. Dev. Ctries. 10, 127–133. 10.3855/jidc.6835 26927452

[B53] Quilici M. L. Robert-Pillot A. Picart J. Fournier J. M. (2005). Pandemic *Vibrio parahaemolyticus* O3:K6 spread, France. Emerg. Infect. Dis. 11, 1148–1149. 10.3201/eid1107.041008 16032794 PMC3371812

[B54] Raghunath P. (2014). Roles of thermostable direct hemolysin (TDH) and TDH-related hemolysin (TRH) in *Vibrio parahaemolyticus*. Front. Microbiol. 5:805. 10.3389/fmicb.2014.00805 25657643 PMC4302984

[B55] Rosec J. P. Simon M. Causse V. Boudjemaa M. (2009). Detection of total and pathogenic *Vibrio parahaemolyticus* in shellfish: comparison of PCR protocols using pR72H or toxR targets with a culture method. Int. J. Food Microbiol. 129, 136–145. 10.1016/j.ijfoodmicro.2008.11.017 19106014

[B56] Su Y. C. Liu C. (2007). *Vibrio parahaemolyticus*: a concern of seafood safety. Food Microbiol. 24, 549–558. 10.1016/j.fm.2007.01.005 17418305

[B57] Sun H. Li Y. Shi X. Lin Y. Qiu Y. Zhang J.. (2015). Association of CRISPR/Cas evolution with *Vibrio parahaemolyticus* virulence factors and genotypes. Foodborne Pathog. Dis. 12, 68–73. 10.1089/fpd.2014.1792 25455966

[B58] Velazquez-Roman J. Leon-Sicairos N. De Jesus Hernandez-Diaz L. Canizalez-Roman A. (2013). Pandemic *Vibrio parahaemolyticus* O3:K6 on the American continent. Front. Cell. Infect. Microbiol. 3:110. 10.3389/fcimb.2013.00110 24427744 PMC3878053

[B59] Wang D. Wang H. Zhou Y. Zhang Q. Zhang F. Du P.. (2011). Genome sequencing reveals unique mutations in characteristic metabolic pathways and the transfer of virulence genes between *V. mimicus* and *V. cholerae*. PLoS ONE 6:e21299. 10.1371/journal.pone.0021299 21731695 PMC3120857

[B60] Williams T. L. Musser S. M. Nordstrom J. L. Depaola A. Monday S. R. (2004). Identification of a protein biomarker unique to the pandemic O3:K6 clone of *Vibrio parahaemolyticus*. J. Clin. Microbiol. 42, 1657–1665. 10.1128/JCM.42.4.1657-1665.2004 15071022 PMC387615

[B61] Wong H. C. Lin C. H. (2001). Evaluation of typing of *Vibrio parahaemolyticus* by three PCR methods using specific primers. J. Clin. Microbiol. 39, 4233–4240. 10.1128/JCM.39.12.4233-4240.2001 11724826 PMC88530

[B62] Wong H. C. Liu C. C. Pan T. M. Wang T. K. Lee C. L. Shih D. Y. (1999). Molecular typing of *Vibrio parahaemolyticus* isolates, obtained from patients involved in food poisoning outbreaks in Taiwan, by random amplified polymorphic DNA analysis. J. Clin. Microbiol. 37, 1809–1812. 10325328 10.1128/jcm.37.6.1809-1812.1999 PMC84956

[B63] Wong H. C. Lu K. T. Pan T. M. Lee C. L. Shih D. Y. (1996). Subspecies typing of *Vibrio parahaemolyticus* by pulsed-field gel electrophoresis. J. Clin. Microbiol. 34, 1535–1539. 8735112 10.1128/jcm.34.6.1535-1539.1996 PMC229056

[B64] Yan W. X. Dai Y. Zhou Y. J. Liu H. Duan S. G. Han H. H.. (2015). Risk factors for sporadic *Vibrio parahaemolyticus* gastroenteritis in east China: a matched case-control study. Epidemiol. Infect. 143, 1020–1028. 10.1017/S0950268814001599 24992005 PMC9507111

[B65] Zhang C. Hu X. Luo J. Wu Z. Wang L. Li B.. (2015). Degradation dynamics of glyphosate in different types of citrus orchard soils in China. Molecules 20, 1161–1175. 10.3390/molecules20011161 25587790 PMC6272633

[B66] Zhang H. Sun S. Shi W. Cui L. Gu Q. (2013). Serotype, virulence, and genetic traits of foodborne and clinical *Vibrio parahaemolyticus* isolates in Shanghai, China. Foodborne Pathog. Dis. 10, 796–804. 10.1089/fpd.2012.1378 23988077

